# Longitudinal study of the influence of lung function on vascular health from adolescence to early adulthood in a British multiethnic cohort

**DOI:** 10.1097/HJH.0000000000001455

**Published:** 2017-07-18

**Authors:** Yao Lu, Lum Sooky, Maria João Silva, Oarabile R. Molaodi, Alexis Karamanos, J. Kennedy Cruickshank, Seeromanie Harding

**Affiliations:** a Division of Diabetes and Nutritional Sciences, Cardiovascular Medicine Group, King's College London, London, UK; b Centre of Clinical Pharmacy, The Third Xiangya Hospital, Central South University, Changsha, China; c Respiratory, Critical Care and Anaesthesia section, University College London Great Ormond Street Institute of Child Health, London, UK; d MRC/CSO Social and Public Health Sciences Unit, Institute of Health and Wellbeing, University of Glasgow, Glasgow, Scotland; e Department Epidemiology and Health, ESRC International Centre for Lifecourse Studies in Society and Health, University College London, London, UK

**Keywords:** augmentation index, blood pressure, forced expiratory volume, longitudinal studies, pulse wave velocity

## Abstract

**Background:**

Vascular and lung function develop and decline over the life course; both predict cardiovascular events and mortality but little is known of how they develop over time. We analysed their relationship in a multiethnic cohort study to test whether lung function from early adolescence to young adulthood affected vascular indices.

**Methods:**

‘DASH’ (http://dash.sphsu.mrc.ac.uk) included 6643 children aged 11–13 years in 2003; a representative 10% sample (*n* = 665) participated in a pilot follow-up in 2013. Psychosocial, anthropometric, blood pressure (BP), and lung function measures were collected in both surveys; aortic pulse wave velocity (PWV) and augmentation index (AIx) were measured at aged 21–23 years. Relationships between forced expiratory volume Z-scores in 1 s (zFEV_1_), after global initiative-ethnic adjustments and BP, PWV, and AIx were tested in linear regression and general estimating statistical models.

**Results:**

In total, 488 people with complete data were included. At 11–13 years, SBP was positively associated with zFEV_1_ (coefficient = 1.90, 95% confidence interval 1.11–2.68, *P* < 0.001); but not at 21–23 years. The 10-year increase in zFEV_1_ was associated with rise in SBP (1.38, 0.25–1.51, *P* < 0.05) in mixed effect models adjusted for age, sex, ethnicity, waist to height ratio, employment, reported racism, smoking, and alcohol use but DBP change was unrelated. In fully adjusted models, neither PWV nor central AIx were associated with zFEV_1_ at 11–13 years or 21–23 years (*P* > 0.05).

**Conclusion:**

Forced expiratory volume change is positively and independently associated with SBP change from adolescence to young adulthood, suggesting earlier lung function plays important roles in SBP development. Vascular indices were unrelated to lung function or its change.

## INTRODUCTION

In adulthood, impaired lung function is inversely related to the incidence of stroke [1], myocardial infarction, and cardiovascular mortality [2], independently of (after adjustment for) traditional cardiovascular risk factors in middle-age populations. Given lung function's significance for adult vascular health, surprisingly few studies have examined the interrelationships of their developmental trajectories from childhood. Lung development starts *in utero* and continues through adolescence and early adulthood [3,4], which raises the question whether suboptimal lung growth and development could affect later vascular function and blood pressure (BP).

Whether associations between low lung function and incidence of vascular events could be related to or mediated by its link with BP and vessel dysfunction remain unclear. The inverse association between lung function and BP has been reported in several adult studies [5–7]. In middle aged (45–59 years) and elderly (>60 years) population, lung function parameters measured at age 40 were stronger predictors of arterial stiffness [aortic pulse wave velocity (PWV) and pressure or flow wave reflection measured as augmentation index (AIx)] than lung function at age 60 [8–10]. Furthermore, lung function and vascular function may track each other through convergent inflammatory or metabolic pathways throughout life, or genetic susceptibility. Such low-grade systemic inflammation is indicated by higher levels of C-reactive protein, fibrinogen, and other systemic inflammatory markers [11].

There is increasing evidence of ethnic differences in the development of both lung function and BP. Lung function is lower among South Asians and Black African origin children compared with White European peers even after adjustment for age, sex, and height, findings which led to the recent development of ethnic specific equations [12].

A faster rise of BP has been observed among African origin children in the United States [13] and among all ethnic minority groups in the United Kingdom [14,15] than their White counterparts. These studies signal that ethnic differences in growth velocities in early life and postnatally are important determinants of cardiorespiratory development.

To date, information is lacking on the determinants of cardiorespiratory development from an ethnically diverse population in early adulthood, a time when physical health is at its peak and when early signs of disease begin to appear. Studies that tracked growth from childhood and collected biomarkers and vascular measures are mainly of White Europeans with few in Europe varying in ethnic make-up [16,17]. The Determinants of Adolescent Social Well-Being and Health (DASH) study, established in 2002, has followed health and social exposures of over 6000 young Londoners (United Kingdom), including 80% ethnic minorities, over the last 12 years. A pilot follow-up study of the cohort, now in their early 1920s, was recently completed. We used the DASH study to examine potential associations between lung function and BP, PWV, and AIx from early adolescence to early 1920s, and whether any observed association is influenced by sex, health behaviours, and socioeconomic circumstances (SEC).

## METHOD

### Details of the Determinants of Adolescent Social Well-Being and Health study can be found elsewhere

In 2002–2003, a total of 6643 students, aged 11–13 years, from 51 secondary schools in 10 London boroughs, were recruited [18]. In 2005–2006, 4785 (88% of children in 49 schools, 72% of the initial cohort), participated in the first follow-up aged 14–16 year. A 10% subsample (*N* = 665, 97% of those contacted) participated in the pilot follow-up, completed in March 2014. The subsample consisted of 107 White UK, 102 Black Caribbean, 132 Black African, 99 Indian, 111 Bangladeshi or Pakistani, and 115 other (mainly mixed) ethnicities. We first located the sample and 81% (5414 of 6643) of the cohort was traced through friendship networks, social media, and community campaigns. We then randomly selected 100 (50 per sex) per ethnic group, and pragmatically attempted, by repeated random selection, to ensure representation across the London boroughs, schools, and SEC at 11–13 years.

The study was approved by the Multicentre Research Ethics Committee and additionally at age 21–23 years from National Health Service Local Research Ethics Committees. Written informed consent was obtained from all participants. Ethnicity was self-reported, checked against reported parental ethnicity, and grandparents’ country of birth. Bangladeshis and Pakistanis were combined because of small sample sizes.

### Spirometry data management and exclusion criteria

Spirometric assessments were undertaken using a portable Micro-plus spirometer (Micro Medical Ltd., Kent, UK) at 11–13 years and 21–23 years in accordance with published guidelines [19]. Details of data quality and acceptability from 11–13 years survey have been published [20]. Data collected at 21–23 years were overread by a senior respiratory physiologist (University College London Great Ormond Street Institute of Child Health) to ensure appropriate quality control.

As the cohort is a healthy community sample of young people, the forced expired volume in 1 s (FEV_1_) provides the optimal survey measure of airway caliber, with lower values or rapid decline in FEV_1_ being associated with higher morbidity or unfavourable prognosis [21]. Missing data, implausible values or extreme outliers (−3< or >3 Z-scores) for height and FEV_1_, (e.g.: when FEV_1_ between 11–13 years and 21–23 years differed by <100 ml), or those with current asthma were excluded (*n* = 177; Figure S1).

After adjusting for sex, age, and standing height, FEV_1_ was expressed as Z-scores using the Global Lung Function Initiative (GLI-2012) ethnic-specific equations [12]. As reliable spirometric equations for South Asians are lacking, the GLI-Black equation, which provided a better approximation for interpreting South Asian data was used instead [22]. Within a healthy population, the mean (SD) FEV_1_ Z-scores would approximate 0(1). A Z-score difference in FEV_1_ is equivalent to 12% of predicted FEV_1_ at these ages.

### Anthropometry, blood pressure, arterial stiffness, and wave reflection measurements

Field staff was trained for 1 week prior to the start of fieldwork, and were recertified at 6-month intervals. Equipments were calibrated regularly by the field supervisors. During adolescence, assessments were conducted in schools and at 21–23 years, in community locations (e.g. local general practitioner's surgeries, local community pharmacies, survey clinic rooms at Kings College London).

Height was measured using portable stadiometers (Leicester, UK), to the nearest 0.1 cm and weight using Salter electronic scales, to the nearest 0.1 kg. Waist circumference (cm) was measured midway between the 10th rib and the top of the iliac crest, and 0.5 cm subtracted to correct for measurement over T-shirt or vest. The mean of two duplicate measures was derived for the waist to height ratio (WHtR). SBP, and DBP were measured at both time points using validated OMRON M5-I semiautomatic devices (Kyoto, Japan), and appropriately sized cuffs, after participant had sat quietly for a timed 5 min, with more than 1 min between three readings. The mean of second and third readings was analysed, as previously reported [14]. At 21–23 years, central SBP (cSBP), pulse, PWV, central AIx (AIxao), and brachial BP were also measured using the Arteriograph 24-h device (TensioMed, Budapest, Hungary), previously calibrated and standardized [23].

### Social exposures

A self-administered questionnaire measured smoking, alcohol consumption, racism, and SEC at 11–13 years and 21–23 years. Reported racism was assessed using validated questions on unfair treatment on grounds of race, skin colour, country of birth, or religion in various locations (school, neighbourhoods, and work) [24]. In adolescence, SEC was assessed using parental employment and the Family Affluence Scale [25] based on number of cars, computers, and holidays. In adulthood, SEC was measured using own education and employment.

### Statistical analyses

Continuous variables (SBP, DBP, WHtR, FEV) did not require transformation as the Shapiro–Wilk test indicated normal distributions. For BP, we first examined the cross-sectional associations between FEV_1_ Z-score and BP at 11–13 years and at 21–23 years, using linear regression models. We then used linear mixed models to examine the association between the change in FEV_1_ and the change in BP, between 11–13 years and 21–23 years. In these latter analyses zFEV_1_, sex, and ethnicity at 11–13 years were added as fixed covariates, and change in zFEV_1_ and remaining social exposures were added as time varying covariates. All variables were added stepwise and two models (model with age, sex, ethnicity, WHtR, and the model with further adjustment for all social exposures) were presented. For outcomes that were measured only at 21–23 years (cSBP, PWV, Aix, and FEV1), linear regression models were used to examine associations between these outcomes and forced expiratory volume Z-scores in 1 s (zFEV_1_) at 11–13 years and at 21–23 years. Two sets of models were run. The first set was with zFEV1 at 11–13 years to examine, whether at this critical period of adolescence, there was an association with these outcomes at 21–23 years. The second repeated the analyses with zFEV_1_ at 21–23. All the analyses were performed STATA 13 (Stat Corp., College Station, Texas, USA). *P* < 0.05 were considered to be statistically significant.

## RESULTS

### Study population

After exclusions, 488 subjects (53.1% males) were included in the analyses. At age 11–13 years, the longitudinal sample's profile for most anthropometric parameters, BP and FEV_1_ did not vary significantly by gender (Table 1). At 21–23 years, men were taller and had higher BP, brachial SBP, cSBP, and PWV but a lower heart rate and AIxao. Mean zFEV_1_ for all ethnic groups and at both ages were within ± 0.5 Z-scores; Table S1). The 10-year increase in height, SBP, and DBP was greater for Whites than for other ethnic minority groups (Table S2). At 21–23 years, Whites also had a higher PWV and lower AIxao (Table S1).

### Forced expiratory volume Z-scores in 1 s and blood pressure

**FIGURE 1:**
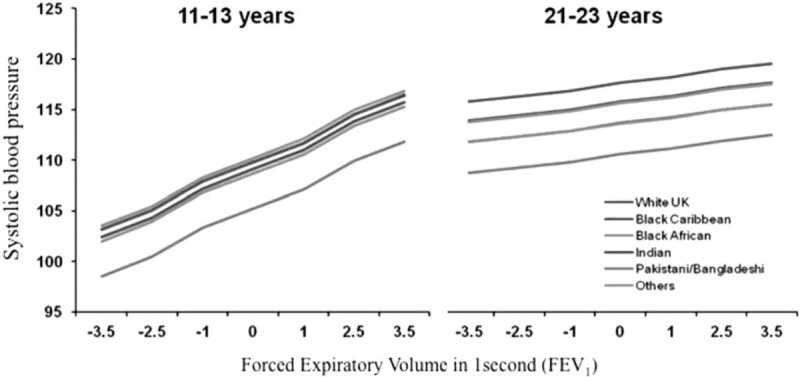
Predicted mean systolic blood pressure by zFEV1 (superscript for footnote) at 11–13 years and at 21–23 years, derived from linear regression models adjusted for age, sex, ethnicity, and waist to height ratio. DASH, The Determinants of Adolescent Social Well-being and Health; zFEV1, forced expiratory volume Z-scores in 1 s.

Analyses of the cross-sectional data at 11–13 years showed that zFEV_1_ was positively and independently associated with SBP at 11–13 year (Table 2). Unadjusted, an increase in 1 zFEV_1_ score was associated with +2.1 [95% confidence interval (CI) 1.3–2.9] mmHg rise in SBP. Adjusting for age, sex, ethnicity, and WHtR reduced this effect a little [+1.90 (1.11–2.68) mmHg]. There was no significant change on further adjustment of racism, parental employment, current smoking, and alcohol use. zFEV1 was not associated with DBP at 11–13 years. At 21–23 years, zFEV1 was positively but not significantly associated with SBP (Table 2). Figure 1 shows the predicted means by ethnicity, derived from the core model, adjusted for age, sex, and WHtR. A similar pattern was observed across the ethnic groups, with a stronger association between zFEV1 and SBP at 11–13 years than 21–23 years. zFEV_1_ was not associated with DBP (Table S3).

In analysis of the longitudinal data, changes in zFEV1 between 11–13 years and 21–23 years were associated with SBP between 11–13 years and 21–23 years (Table 2). Adjusting for age, gender, ethnicity, changes in WHtR were positively associated with SBP, the pooled average being +1.38 (0.3–1.6) mmHg, unaltered by lifestyle and social exposures. Changes in zFEV_1_ were not associated with DBP between 11–13 years and 21–23 years (Table S4).

### The associations between forced expiratory volume Z-scores in 1 s and central SBP, pulse wave velocity, and central augmentation index

zFEV_1_ at 11–13 years or 21–23 years was not significantly associated with cSBP, PWV, or AIx at 21–23 years (Table 3). PWV was significantly associated with sex (women lower), age, WHtR, and heart rate, the latter two also reducing AIx, which was unaffected by age or social exposures.

## DISCUSSION

To the best of our knowledge, this is the first longitudinal study with a diverse ethnic composition, as yet from a pilot follow-up, to report an association between lung function and SBP between adolescence and early adulthood. The longitudinal changes in lung function are positively associated with SBP during this part of the life course when considerable growth occurs. The mechanisms underlying these associations are not well understood but could illustrate how greater lung growth marginally, but likely reversibly, impairs venous return to the heart over this growth period, with a reflex rise in SBP.

Normal developmental trajectory for lung function shows that it increases in childhood, peaks in young adulthood and then gradually decreases with age [12,26,27]. A US-based longitudinal study (*N* = 508) showed that a decline in lung function from average peak age (29.4 years) to 35 years significantly predicted incidence of hypertension between mean ages 35–45 years. In correspondence with DASH findings, the cross-sectional Wheezing Illnesses Study Leidsche Rijn (WHISTLER) study of 5-year-olds (*N* = 382) in the Netherlands showed a positive relationship between FEV_1_ and SBP (+4.8 mmHg/l, 95% CI: −0.3 to 10.0). WHISTLER also showed a positive relationship with DBP (+4.6 mmHg/l, 95% CI: −0.2 to 9.4) adjusted for age, sex, weight, and height [28]. The results of WHISTLER and DASH, though discrepant in relation to DBP, suggest that the positive relationship between BP and lung function is normal physiological development in childhood and in early adolescence. As the WHISTLER result suggests a litre change in FEV_1_ was associated with ∼5 mmHg in SBP change in a 5-year-old child, when mean (SD) FEV_1_ would be only 1.3(0.2) l, a change of 200 ml (1 SD) in FEV_1_ would be associated with a 1 mmHg increase in SBP. Similar results were observed here in DASH.

As with lung function, the association between PWV and lung function has not been studied before in healthy children. A cross-sectional study of asthmatic children (aged 6.1–15.3 years) in Switzerland showed that carotid-femoral PWV was inversely associated (*r*^2^=0.20, *P* = 0.004) with FEV_1_[29]. It is noteworthy that the effect size of about +2 mmHg increase in SBP these studies with 1 Z-score of FEV1 is comparable across these studies of 5-year-old and of 11–13 years in DASH. Perhaps in contrast to the DASH findings, a cross-sectional community-based study of 249 8-year-old children in Australia showed FEV_1_ was inversely associated with carotid (not brachial, as here) AIx75 (coefficient = −0.17, *P* = 0.03) and forced vital capacity (coefficient = −0.29, *P* < 0.001) [30].

Our results showed that lung function is associated with SBP at 11–13 years, and the change in lung function from 11 to 23 years is associated with the change in SBP. At 21–23 years lung function was not associated with SBP or PWV. There are very few studies that have examined cardiorespiratory health in healthy young adults. Garcia-Larsen *et al.*[31] showed no association between lung function and hypertension in 25-year-olds. We are not aware of any studies that have examined the association of lung function and PWV in young adults. One possible explanation for the lack of association between lung function and PWV is that PWV was measured only at 21–23 years (not at 11–13 years). At 11–23 years, lung and vascular function are expected to be within a normal range. Thus, these findings cannot be extrapolated to older age when other risk factors can affect both parameters. In addition, pollutants can deeply influence BP [32] as well as lung function [33], even in adolescence. The impact of pollution is the focus of separate analyses.

### Strengths and weaknesses of study

Social exposures, for example, deprivation, influence growth [34], which in turn influences cardiorespiratory development. The studies cited above did not examine potential moderating influences from social exposures. DASH contains information on socioeconomic disadvantage and behaviours, which enabled an examination of their association on the zFEV_1_-cardiovascular disease measure. DASH contains a diverse sample, has high retention rates and low item-nonresponse, mainly because of enormous community support. Lung function was not measured at the second follow-up of the cohort at 14–16 years, which could have provided valuable insight into the pattern of changing associations with BP in adolescence. The lack of data before age 11 years and the relatively small sample sizes for the ethnic groups in the pilot follow-up are limitations. Prior to DASH a large-scale study with an explicit focus on ethnicity had not been attempted. The use of the GLI-2012 ethnic-specific equations rather than absolute FEV also strengthened the analysis. These robust equations have been shown to be appropriate for London school children [35] and the small offset (≤0.5 Z-scores) in mean FEV_1_ in all ethnic groups observed in this study suggest a sample size issue. Quanjer *et al.* suggested that a dataset of at least 300 (150 men and 150 women) would be required to validate reference values to avoid spurious differences because of sampling error [36]. Despite relatively small numbers, these findings in a tight age range provide strong justification for future studies on the degree and how childhood and adolescent lung function influence vascular development to improve understanding of the developmental interaction of cardio-respiratory systems.

## ACKNOWLEDGEMENTS

We acknowledge the invaluable support of participants and their parents, the Participant Advisory Group, schools, civic leaders, local GP surgeries and community pharmacies, the Clinical Research Centre at Queen Mary University of London, the Clinical Research Facility at University College Hospital, the survey assistants and nurses involved with data collection, Rachel Bonner, senior respiratory physiologist (UCL GOS ICH) for spirometry training and over-reading of data for quality control, the Primary Care Research Network, and Professor Sanders and J.K.C at the Diabetes and Nutritional Sciences Division at Kings College London for hosting the feasibility study. H.S. is the Principal Investigator of DASH. All authors contributed to study design, analyses, and writing of the article. The study was funded by the MRC (MC_U130015185/MC_ UU_12017/13), Chief Scientist Office (SPHSU13), North Central London Research Consortium, and the Primary Care Research Network.

### Conflicts of interest

There are no conflicts of interest.

## Supplementary Material

Supplemental Digital Content

## Figures and Tables

**TABLE 1:** Sample characteristics by sex

Variables	11–13 years		21–23 years	
	Male	Female	Male	Female
Age (years)	12.6 (12.5,12.7)	12.5 (12.4,12.6)	22.9 (22.8,23.0)	22.7 (22.6,22.8)
Height (cm)	155.0 (153.7,156.3)	155.7 (154.7,156.7)	176.1 (175.2,176.8)	163.7 (162.8,164.5)
Waist to height ratio	0.40 (0.41,0.44)	0.43 (0.42,0.44)	0.48 (0.47,0.49)	0.49 (0.08,0.50)
SBP (mmHg)	109.2 (107.94,110.50)	107.2 (106.0,108.3)	120.2 (118.9,121.4)	107.1 (105.8,108.4)
DBP (mmHg)	66.5 (65.5,67.5)	66.7 (65.8,67.6)	73.5 (72.5,74.5)	71.4 (70.4,72.4)
FEV_1_ (l)	2.4 (2.3,2.5)	2.3 (2.3,2.4)	3.9 (3.7,4.1)	2.9 (2.8,2.9)
zFEV_1_	−0.22 (1.08)	−0.32 (1.09)	−0.43 (0.98)	−0.43 (1.01)
Pulse (beats/min)			66.4 (65.0,67.7)	71.3 (69.8,72.7)
zFEV_1_ (%)				
−1 to +1 Z score	62.1 (56.3,68.1)	61.6 (55.2,68.1)	62.5 (56.6,68.3)	65.8 (59.4,72.1)
Less than 1 Z score	24.5 (19.4,29.7)	28.8 (22.7,34.8)	28.6 (23.2,34.1)	26.5 (20.6,32.4)
>1 Z score	13.4 (9.3,17.5)	9.6 (5.7,13.5)	8.9 (5.5,12.3)	7.8 (4.2,11.3)
Brachial SBP (mmHg)			121.8 (120.3,123.3)	112.7 (111.0,114.4)
^a^cSBP (mmHg)			109.6 (108.2,111.0)	103.6 (102.2,105.0)
PWV (m/s)^b^			7.4 (7.19,7.6)	6.8 (6.6,6.9)
AIxao (%)			12.1 (11.0,13.3)	15.6 (14.1,17.0)
Current smoker (%)	0.01 (−0.001,0.2)	0.02 (0.001,0.03)	32.7 (27.1,38.3)	37.4 (31.0,43.9)
Current alcohol user (%)	25.7 (20.4,30.9)	24.7 (18.9,30.4)	53.2 (47.2,59.2)	61.2 (54.7,67.7)
Reported racism (%)	20.4 (15.6,25.3)	20.1 (14.8,25.4)	43.9 (37.9,49.8)	43.4 (36.8,50.0)
Parental/own employment (%)	75.8 (70.7,81.0)	77.6 (72.1,83.2)	48.3 (42.3,54.3)	52.5 (45.9,59.2)
Family Affluence Scale^c^				
≥3	53.5 (47.5,59.5)	53.0 (46.3,59.6)		
1–2	27.5 (22.4,32.9)	29.7 (23.6,35.8)		
Completed a university degree (%)			40.9 (35.0,46.8)	57.5 (51.0,64.1)

The Determinants of Adolescent Social Well-Being and Health study. Values are mean (95% CI) or percentage (95% CI). Z score of FEV1 are mean (SD). AIxao, central augmentation index; CI: confidence interval; cSBP, central SBP; PWV, pulse wave velocity; zFEV_1_, forced expiratory volume Z-scores in 1 s.

^a^cSBP, central SBP.

^b^PWV, pulse wave velocity.

^c^Family affluence scale comprises of number of holidays last year, computers, cars, or vans.

**TABLE 2:** Cross-sectional and longitudinal associations between forced expiratory volume in 1 s and SBP from early childhood to young adults

Variables	SBP at 11–13 years^a^	SBP at 21–23 years^a^	Δ SBP
zFEV_1_	2.1 (1.3,2.9)^b^	0.5 (−0.3,1.4)	0.02 (−1.2,1.1)
ΔzFEV_1_			1.4 (0.3,2.5)^c^
Ethnicity (White UK: ref)			
Black Caribbean	−0.3 (−3.2,2.5)	−1.7 (−4.8,1.4)	−0.8 (−3.3,1.6)
Black African	−0.1 (−2.9,2.7)	−2.0 (−5.1,1.1)	−0.8 (−3.2,1.6)
India	−1.4 (−4.4,1.6)	−4.0 (−7.2,−0.8)^c^	−2.5 (−5.0,0.0)
Pakisitani/Bangladeshi	−5.4 (−8.5,−2.5)^b^	−6.8 (−10.2, −3.4)^b^	−6.4 (−9.0, −3.8)^b^
Others	−1.0 (−3.7,1.7)	−3.8 (−6.8, −0.8)^c^	−2.4 (−4.8, −0.1)^c^
Female	−1.6 (−3.2,0.1)	−13.9 (−15.6, −12.1)^b^	−7.7 (−9.1, −6.3)
Age	2.9 (1.2,4.3)^b^	0.3 (−0.9,1.5)	0.4 (0.3,0.6)^b^
Waist to height ratio	26.9 (12.4,41.4)^b^	31.9 (20.0,43.9)	26.4 (16.4,36.3)^b^
Reported racism (no: ref)			
Yes	−1.3 (−3.4,0.8)	0.2 (−2.0,1.6)	−0.9 (−4.6,2.8)
Parental/own employment (yes: ref)			
No	−0.6 (−2.9,1.7)	−1.0 (−3.1,1.1)	−0.2 (−1.8,1.3)
Current smoking (no: ref)			
Yes	−6.3 (−13.3,0.6)	0.2 (−2.1,2.5)	2.5 (0.4,4.6)^c^
Alcohol use (No: ref)			
Yes	−1.3 (−3.4,0.9)	0.3 (−1.9,2.5)	0.8 (−2.4,0.8)

The Determinants of Adolescent Social Well-Being and Health study. zFEV_1_, lifestyle factors (alcohol and smoking), lifestyle factors, employment, reported racism.

^a^Linear models adjusted for age, sex, ethnicity and waist to height ratio and reported racism, employment, lifestyle factors (smoking and alcohol use; 11–13 years *R*^2^ = 0.17, 21–23 years *R*^2^ = 0.36).

^b^Data were statistically significant at *P* < 0.001.

^c^Data were statistically significant at *P* < 0.05.

**TABLE 3:** The influence of forced expiratory volume in 1 s from early childhood to young adults on central blood pressure, pulse wave velocity, and augmentation index at 21–23 year

Variables	cSBP	PWV	AIXao
zFEV_1_ at 21–23 years	0.4 (−2.1,3.0)	0.03 (−0.2,0.1)	0.2 (−1.1,1.5)
zFEV_1_ at 11–13 years	−0.7 (−3.3,1.8)	0.005 (−0.2,0.2)	−0.5 (−1.7,0.7)
Age	−2.6 (−6.6,1.5)	0.7 (0.2,0.7)^a^	−1.6 (−3.5,0.4)
Female	−5.5 (−9.5,−1.5)^a^	−0.7 (−1.0,−0.4)^a^	2.3 (−0.3,4.9)
Ethnicity (White UK: ref)			
Black Caribbean	−2.4 (−8.5,3.6)	−0.1 (−-0.6,0.3)	0.1 (−3.1,3.3)
Black African	1.4 (−5.2,8.0)	−0.3 (−0.7,0.2)	3.0 (−0.3,6.3)
India	−5.0 (−11.5,1.5)	−0.2 (−0.7,0.2)	1.5 (−2.0,4.9)
Pakisitani/Bangladeshi	−3.4 (−11.4,4.6)	−0.5 (−1.0,0.1)	3.3 (−0.5,7.1)
Others	−1.2 (−7.1,4.8)	−0.3 (−0.8,0.1)	0.9 (−2.1,4.0)
Waist to height ratio	65.8 (27.6,104.0)^a^	2.9 (0.4,5.4)^b^	−0.3 (−0.5, −0.2^a^
Pulse	−0.03 (−0.2,0.1)	0.04 (0.03,0.05)^a^	−0.3 (−0.4, −0.2)^a^
brSBP	–	0.01 (−0.00,0.02)	0.1 (0.04,0.2)^c^

The Determinants of Adolescent Social Well-Being and Health study. Linear models adjusted for age, sex, ethnicity and waist to height ratio, pulse and (brachial blood pressure for PWV and AIXao) at 21–23 years and reported racism, employment, education, smoking and alcohol using at 21–23 years and adolescent adjustment (*R*^2^ = 0.17, 0.29, and 0.23). AIxao, central augmentation index; PWV, pulse wave velocity.

^a^Data were statistically significant at *P* < 0.001.

^b^Data were statistically significant at *P* < 0.05.

^c^Data were statistically significant at *P* < 0.01.

## Reviewers’ Summary Evaluations

### Reviewer 2

The present longitudinal study aims to demonstrate a relationship between vascular and lung function considering that the alterations of both systems can predict cardiovascular events. The most interesting and intriguing finding is that forced expiratory volume modifications are associated with systolic blood pressure values from adolescence (11-13 years) to young adulthood (10 years after). Obviously, the study is descriptive and it is not possible to establish a clear cause-effect relationship, also considering that lung function is not related to aortic pulse way velocity, one of the main determinants of systolic blood pressure. However, the study demonstrates an important relationship between pulmonary and vascular function, which might be of great relevance in the pathogenesis of cardiovascular disease.
