# Supplementary material for: Longitudinal study of the influence of lung function on vascular health from adolescence to early adulthood in a British multiethnic cohort
Source: J Hypertens. 2017 Jul 18;35(11):2185–91. doi: 10.1097/HJH.0000000000001455 (PMC5625963; doi:10.1097/HJH.0000000000001455)

Table S1 Sample characteristics by gender. The Determinants of Adolescent Social well-being and Health study

| Variables | White UK | Black Caribbean | Black African | India | Pakistani/Bangladeshi | Other |
| --- | --- | --- | --- | --- | --- | --- |
|  | N░=░86 | N░=░73 | N░=░88 | N░=░71 | N░=░82 | N░=░88 |
| Boys | 45 | 35 | 46 | 41 | 42 | 50 |
| Age | | | | | | |
| 11–13y | 12.6(12.5,12.7) | 12.6(12.5,12.8) | 12.6(12.5,12.7) | 12.5(12.3,12.6) | 12.4(12.3,12.6) | 12.6(12.5,12.7) |
| 21–23y | 22.7(22.6,22.8) | 22.8(22.6,23.0) | 22.7(22.5,22.9) | 22.9(22.7,23.1) | 22.7(22.6,22.9) | 22.9(22.8,23.1) |
| Height(cm) | | | | | | |
| 11–13y | 155.6(153.6,157.5) | 157.3(155.2,159.5) | 159.4(157.6,161.2) | 151.6(149.7,153.5) | 151.9(149.7,154.1) | 155.6(153.7,157.5) |
| 21–23y | 173.1(171.1,175.0) | 170.6(168.4,172.9) | 172.3(170.4,174.3) | 168.5(166.2,170.7) | 168.1(166.1,170.0) | 170.0(168.2,171.9) |
| Waist-Height Ratio | | | | | | |
| 11–13y | 0.43(0.42,0.44) | 0.426(0.41,0.44) | 0.43(0.41,0.44) | 0.43(0.42,0.45) | 0.42(0.41,0.43) | 0.432(0.42,0.44) |
| 21–23y | 0.47(0.46,0.49) | 0.48(0.46,0.50) | 0.48(0.47,0.50) | 0.49(0.47,0.51) | 0.49(0.48,0.50) | 0.48(0.46,0.49) |
| SBP(mmHg) | | | | | | |
| 11–13y | 109.1(107.14,111.0) | 109.4(106.8,111.9) | 109.6(107.6,111.6) | 108.8(106.7,111.0) | 105.1(102.9,107.3) | 107.9(105.8,110.0) |
| 21–23y | 116.7(113.8,119.6) | 114.6(112.0,117.2) | 114.9(112.5,117.3) | 114.1(111.2,117.0) | 111.9(109.3,114.4) | 113.5(111.2,115.9) |
| DBP(mmHg) | | | | | | |
| 11–13y | 66.6(65.0,68.1) | 67.9(65.8,69.9) | 66.6(65.2,68.0) | 68.0(66.2,69.7) | 65.7(64.1,67.3) | 65.2(63.7,66.8) |
| 21–23y | 73.4(71.6,75.3) | 73.5(71.7,75.2) | 72.9(71.2,74.6) | 72.8(70.9,74.7) | 71.4(69.7,73.1) | 71.4(69.7,73.0) |
| FEV_1_(L) | | | | | | |
| 11–13y | 2.6(2.5,2.7) | 2.4(2.2,2.5) | 2.4(2.3,2.5) | 2.1(2.0,2.3) | 2.3(2.2,2.4) | 2.4(2.3,2.5) |
| 21–23y | 3.9(3.7,4.1) | 3.3(3.1,3.4) | 3.3(3.1,3.4) | 3.2(3.0,3.3) | 3.3(3.2,3.5) | 3.5(3.3,3.6) |
| zFEV_1_ | | | | | | |
| 11–13y | -0.43(1.12) | -0.21(1.07) | -0.28(1.04) | -0.25(0.98) | 0.13(1.05) | -0.51(1.16) |
| 21–23y | -0.52(1.00) | -0.35(0.90) | -0.49(0.95) | -0.46(0.75) | -0.15(1.02) | -0.58(1.21) |
| †cSBP(mmHg) | | | | | | |
| 21–23y | 108.9(106.4,11.4) | 109.1(106.1,112.1) | 107.6(105.2,110.0) | 104.8(102.2,107.3) | 105.2(102.9,107.5) | 104.5(102.3,106.6) |
| Brachial SBP(mmHg) | | | | | | |
| 21–23y | 120.6(117.1,124.0) | 119.8(116.9,122.8) | 118.7(115.5,121.9) | 116.6(113.8,119.4) | 114.4(111.8,117.0) | 116.2(113.7,118.7) |
| Pulse | | | | | | |
| 21–23y | 71.0(68.5,73.6) | 66.6(64.2,69.0) | 65.2(62.7,67.6) | 69.0(66.2,71.8) | 70.6(68.3,72.9) | 68.7(66.4,71.1) |
| PWV(m/s) | | | | | | |
| 21–23y | 7.4(7.0,7.8) | 7.1(6.8,7.4) | 6.87(6.59,7.2) | 7.2(6.9,7.5) | 7.1(6.8,7.5) | 7.1(6.8,7.4) |
| AIXao (%) | | | | | | |
| 21–23y | 11.5(9.6,13.5) | 14.3(12.1,16.5) | 15.4(12.3,18.5) | 12.9(10.7,15.0) | 14.6(12.4,16.9) | 13.2(11.5,15.0) |
| Reported racism (%) | | | | | | |
| 11–13y | 14.0(6.6,21.3) | 20.5(11.2,29.9) | 20.5(12.0,29.0) | 15.5(7.0,24.0) | 26.8(17.2,36.5) | 23.9(14.9,32.8) |
| 21–23y | 22.1(13.3,30.9) | 50.7(39.1,62.3) | 51.1(40.6,61.7) | 40.8(29.30,52.4) | 46.3(35.5,57.2) | 51.1(40.6,61.7) |
| Employment (%) | | | | | | |
| Parental | 87.2(80.1,94.3) | 78.1(68.5,87.7) | 78.4 (69.7,87.1) | 77.5(67.7,87.3) | 52.4(41.5,63.3) | 85.2(77.8,92.7) |
| Own | 53.5(42.9,64.1) | 50.7(39.1,62.3) | 52.3(41.8,62.8) | 50.7(39.0,62.4) | 41.5(30.7,52.2) | 52.3(41.8,62.8) |
| Current smoking (yes) (%) | | | | | | |
| 11–13y | 0.02(-0.001,0.05) | 0.05(0.002,0.1) | 0 | 0 | 0.01(-0.01,0.04) | 0 |
| 21–23y | 29.1(19.4,38.7) | 39.7(28.4,51.1) | 39.8(29.5,50.1) | 33.8(22.7,44.9) | 26.8(17.2,36.5) | 39.8(29.5,50.1) |
| Alcohol Using (yes) (%) | | | | | | |
| 11–13y | 48.8(38.2,59.5) | 38.4(27.1,59.16 | 18.2(10.1,26.3) | 4.2(-0.5,9.00) | 1.2(-1.2,3.6) | 37.5(27.3,47.7) |
| 21–23y | 89.5(83.0,96.1) | 74.0(63.8,84.1) | 52.3(41.8,62.8) | 49.3(37.6,61.0) | 8.3(2.43,14.6) | 65.91(55.9,75.9) |
| Family Affluence Scaleɸ (%) | | | | | | |
| >=3 | 57.0(46.4,67.5) | 53.4(41.9,65.0) | 54.5(44.1,65.0) | 47.9(36.2,59.6) | 43.9(33.1,54.7) | 61.4(51.1,71.6) |
| 1--2 | 30.2(20.5,40.0) | 28.8(18.3,39.3) | 22.7(13.9,31.6) | 31.0(20.1,41.8) | 31.7(21.6,41.9) | 27.3(17.9,36.7) |
| Education (%) | | | | | | |
| Has degree | 40.7(30.2,51.2) | 35.6(24.5,46.7) | 62.5(52.3,72.7) | 63.4(52.1,74.7) | 45.1(34.3,56.0) | 43.2(32.7,53.6) |

Values are mean (95%CI) or percentage (95%CI). zscore of FEV1 are mean(SD).

CI: confidence Interval.

†cSBP, central SBP. *PWV, pulse wave velocity.

ɸ Family Affluence Scale comprises of number of holidays last year

Table S2 The difference in anthropometric measures between 21–23y and 11–13y, by ethnicity

| Variables | White UK | Black Caribbean | Black African | India | Pakistani/Bangladeshi | Other |
| --- | --- | --- | --- | --- | --- | --- |
| Height (cm) | 17.5(15.3,19.7) | 13.3(11.3,15.3) | 13.0(11.0,14.9) | 16.9(14.4,19.3) | 16.2(13.9,18.4) | 14.5(12.6,16.3) |
| Waist-Height Ratio | 0.04(0.03,0.06) | 0.06(0.04,0.07) | 0.06(0.04,0.07) | 0.05(0.03,0.07) | 0.07(0.05,0.08) | 0.05(0.04,0.06) |
| SBP(mmHg) | 7.6(5.0,10.2) | 5.21(1.9,8.5) | 5.3(3.0,7.7) | 5.3(2.6,8.02) | 6.7(3.9,9.6) | 5.5(3.2,8.0) |
| DBP(mmHg) | 6.8(5.0,8.7) | 5.62(3.5,7.8) | 6.3(4.5,8.1) | 4.8(2.6,7.1) | 5.7(3.76,7.7) | 6.1(4.2,8.0) |
| FEV_1_(L) | 1.3(1.1,1.4) | 0.9(0.8,1.1) | 0.9(0.8,1.0) | 1.0(0.9,1.2) | 1.0(0.9,1.2) | 1.1(0.9,1.2) |
| zFEV_1_ | -0.09(-0.24,0.07) | -0.14(-0.32,0.05) | -0.21(-0.37,-0.05) | -0.21(-0.39,-0.03) | -0.28(-0.47,-0.09) | -0.07(-0.23,0.09) |

Table S3 The cross-sectional associations between Forced Expiratory Volume in 1second (zFEV_1_) and DBP at 11–13y and 21–23y separately. Sample characteristics by gender. The Determinants of Adolescent Social well-being and Health study

| Anthropometry, lifestyle, social factors | 11–13y | | 21–23y | |
| --- | --- | --- | --- | --- |
|  | Core modelɸ | +lifestyle and social variableǂ | Core modelɸ | +lifestyle and social variableǂ |
| zFEV_1_ | 0.5(-0.1,1.1) | 0.6(-0.02,1.2) | -0.4(-1.0,0.3) | -0.4(-1.0,0.3) |
| Ethnicity (White UK:ref) | | | | |
| Black Caribbean | 1.2(-1.02,3.5) | 1.0(-1.3,3.2) | -0.04(-2.4,2.3) | 0.3(-2.2,2.7) |
| Black African | 0.1(-2.,2.2) | -0.3(-2.5,1.9) | -0.7(-3.0,1.5) | -0.1(-2.6,2.3) |
| India | 1.1(-1.2,3.4) | 0.7(-1.7,3.1) | -1.3(-3.7,1.1) | -0.7(-3.2,1.8) |
| Pakisitani/Bangladeshi | -0.8(-3.0,1.5) | -1.5(-3.9,1.0) | -2.6(-5.0,-0.3)* | -1.5(-4.2,1.2) |
| Others | -1.3(-3.5,0.8) | -1.3(-3.5,0.8) | -2.4(-4.7,-0.2)* | -2.0(-4.3,0.4) |
| Female | 0.4(-1.0,1.7) | 0.5(-0.8,1.8) | -2.5(-3.9,-1.1) ξ | -2.6(-4.0,-1.2) ξ |
| Age | 0.9(-0.2,2.0) | 1.2(0.1,2.3)* | 0.3(-0.6,1.2) | 0.1(-0.8,1.1) |
| Waist:Height ratio | 32.0(20.4,43.6) ξ | 28.7(17.1,40.2) ξ | 31.3(21.9,40.7) ξ | 30.9(21.4,40.4) ξ |
| Reported racism (no:ref) | | | | |
| Yes |  | -1.0(-2.6,0.7) |  | -0.1(-1.6,1.3) |
| Parental/own employment (yes:ref) | | | | |
| NO |  | 0.6(-1.2,2.4) |  | -1.0(-2.7,0.7) |
| Current smoking (Yes: ref) | | | | |
| No |  | -3.5(-9.0,2.1) |  | 1.5(-0.3,3.4) |
| Alcohol use (Yes:ref) | | | | |
| NO |  | -0.5(-2.3,1.2) |  | 1.4(-0.3,3.2) |

*Data were statistically significant at P░<░0.05; #Data were statistically significant at P░<░0.01; ξData were statistically significant at P░<░0.001.

ɸ Core model: adjusted for age, gender, ethnicity and waist:height ratio at 11–13y and 21–23y separately (11–13y R^2^=0.09, 21–23y R^2^=0.12)

ǂFinal model: core model +reported racism + employment +lifestyle factors (alcohol and smoking) (11–13y R^2^=0.13, 21–23y R^2^=0.14).

Table S4 The influence of Forced Expiratory Volume in 1second (zFEV_1_) from early childhood to young adults on DBP. Sample characteristics by gender. The Determinants of Adolescent Social well-being and Health study

| Anthropometry, lifestyle, social factors | Difference from baseline mean diastolic blood pressure | | |
| --- | --- | --- | --- |
|  | Core model | +Lifestyle variables† | +Social variables‡ |
| Change in zFEVs between baseline (11–13y) and follow up (21–23y) | | | |
| ΔzFEV_1_ | -0.2(-1.0,0.7) | -0.2(-1.0,0.7) | -0.2(-1.0,0.7) |
| zFEV_1_ at baseline | 0.4(-0.5,1.2) | 0.4(-0.5,1.2) | 0.4(-0.5,1.3) |
| Female | -1.1(-2.2,-0.004)* | -1.0(-2.1,0.03) | -1.0(-2.1,0.05) |
| Ethnicity (White UK:ref) | | | |
| Black Caribbean | 0.6(-1.3,2.5) | 0.7(-1.2,2.5) | 0.7(-1.2,2.6) |
| Black African | -0.3(-2.1,1.4) | -0.2(-2.1,1.6) | -0.2(-2.0,1.7) |
| India | -0.2(-2.1,1.7) | -0.06(-2.0,1.9) | -0.02(-2.0,1.9) |
| Pakisitani/Bangladeshi | -1.8(-3.6,0.1) | -1.8(-3.7,0.2) | -1.7(-3.7,0.3) |
| Others | -1.9(-3.7,-0.1)* | -1.7(-3.5,0.06) | -1.7(-3.5,0.2) |
| Time-dependent variables | | | |
| ΔAge | 0.4(0.3,0.5) ξ | 0.3(0.2,0.4) ξ | 0.3(0.2,0.4) ξ |
| Δwasit:height ratio | 29.9(22.3,37.5) ξ | 29.5(22.0,37.1) ξ | 29.5(22.0,37.1) ξ |
| Current smoking (No: ref) | | | |
| Δyes |  | 2.3(0.75,3.85)# | 2.3(0.74,3.9)# |
| Acohol use (No:ref) | | | |
| ΔYes |  | 0.3(-0.9,1.4) | 0.3(-0.9,1.4) |
| Employment (Yes:ref) | | | |
| NO |  |  | 0.1 (-1.0,1.3) |
| Racism (no:ref) | | | |
| Yes |  |  | -0.4(-1.4,0.7) |

*Data were statistically significant at P░<░0.05; #Data were statistically significant at P░<░0.01; ξData were statistically significant at P░<░0.001.

Core model: adjusted for age, gender, ethnicity, waist:height ratio and baseline zFEV_1_.

† Core model + lifestyle factors (smoking and alcohol use)

‡Core model +lifestyle factors+ employment +reported racism.

Figure S1 A flow diagram of participant enrollment.


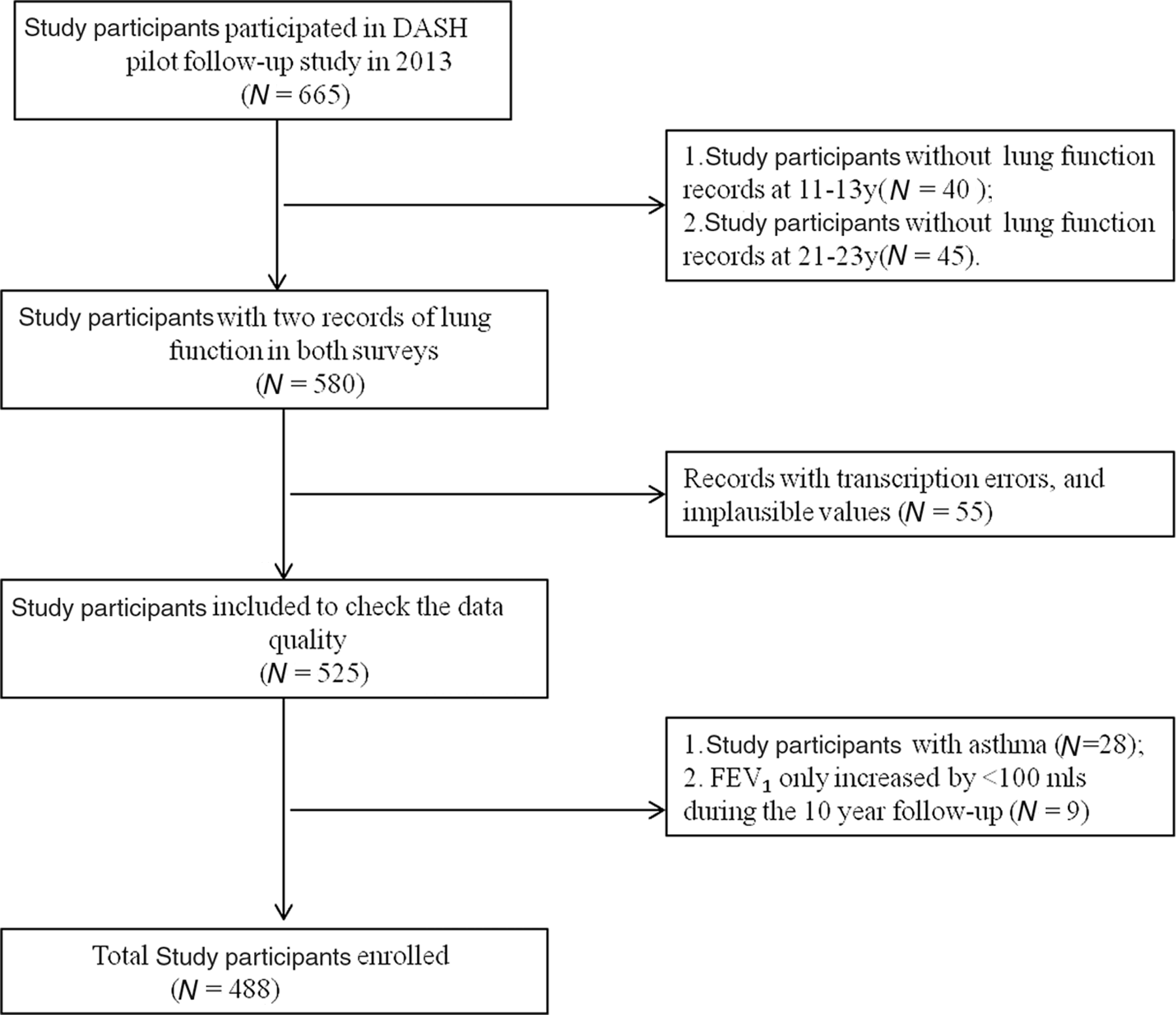

Supplement: Supplemental Digital Content [file jhype-35-2185-s001.doc]
